# Jasmonate: A Hormone of Primary Importance for Temperature Stress Response in Plants

**DOI:** 10.3390/plants12244080

**Published:** 2023-12-06

**Authors:** Meiling Wang, Xiulan Fan, Fei Ding

**Affiliations:** School of Life Sciences, Liaocheng University, Liaocheng 252000, China; 17861828160@163.com

**Keywords:** cold stress, heat stress, temperature stress, jasmonates

## Abstract

Temperature is a critical environmental factor that plays a vital role in plant growth and development. Temperatures below or above the optimum ranges lead to cold or heat stress, respectively. Temperature stress retards plant growth and development, and it reduces crop yields. Jasmonates (JAs) are a class of oxylipin phytohormones that play various roles in growth, development, and stress response. In recent years, studies have demonstrated that cold and heat stress affect JA biosynthesis and signaling, and JA plays an important role in the response to temperature stress. Recent studies have provided a large body of information elucidating the mechanisms underlying JA-mediated temperature stress response. In the present review, we present recent advances in understanding the role of JA in the response to cold and heat stress, and how JA interacts with other phytohormones during this process.

## 1. Introduction

Plants are unable to move and thus have to cope with various adverse environmental factors, such as extreme temperatures, drought, salinity, and heavy metal toxicity [1,2]. Being a critical environmental factor, temperature plays a dominant role in plant growth and development [3], and it determines the geographical distribution of plant species [4]. As global climate change intensifies, the magnitude and frequency of extreme temperatures are increasing. Extreme temperatures cause various forms of damage at different stages during plant growth and development. The general consequences of heat and cold stress include impaired photosynthesis, excessive accumulation of reactive oxygen species, broken plasma membranes, and altered phytohormone levels [5,6,7,8,9,10,11]. Eventually, heat and cold stress inhibit plant growth and cause losses in crop yields, posing a serious threat to food security [12,13,14,15]. A survey showed that wheat, rice, maize, and soybean yields would decrease by 6.0%, 3.2%, 7.4%, and 3.1% on average, respectively, if the global temperature rises by 1 °C [4,16]. Low temperatures adversely affect plant growth and development and reduce crop production [12,17,18,19]. For instance, it has been estimated that in temperate and high-elevation regions, cold stress accounts for 30–40% of yield losses in rice [4,20].

Unlike animals, plants are sessile organisms and thus are unable to escape unfavorable temperature conditions. Instead, plants have evolved a set of sophisticated strategies enabling them to survive under temperature stress. Plant hormones play a vital role in the initiation of temperature stress response by integrating temperature stimulus and endogenous signals. For instance, jasmonates (JAs), abscisic acid (ABA), and brassinosteroid (BR) positively regulate plant response to both heat and cold stress [21,22,23,24,25,26]. Jasmonates (JAs) are a typical class of phytohormones. The term “jasmonates” generally refers to jasmonic acid and its derivatives, typically including jasmonyl isoleucine (JA-Ile) and methyl jasmonate (MeJA) [27]. In addition to its well-known role in growth and development, and in defense against pathogen attack and insect herbivory [28,29,30,31,32], a growing number of studies have highlighted the vital role of JA in the response to a variety of abiotic stresses, including drought, salinity, heat, and cold stress response [33,34,35,36,37,38,39,40,41,42,43]. In the present review, we present recent findings regarding the role of JA in the response to cold and heat stress, and we elucidate the major components in the JA signaling pathway that are involved in cold and heat stress response. We also summarize JA’s interactions with other phytohormones in the response to cold and heat stress and propose future research directions.

## 2. JA Biosynthesis and Signaling

**Figure 1:**
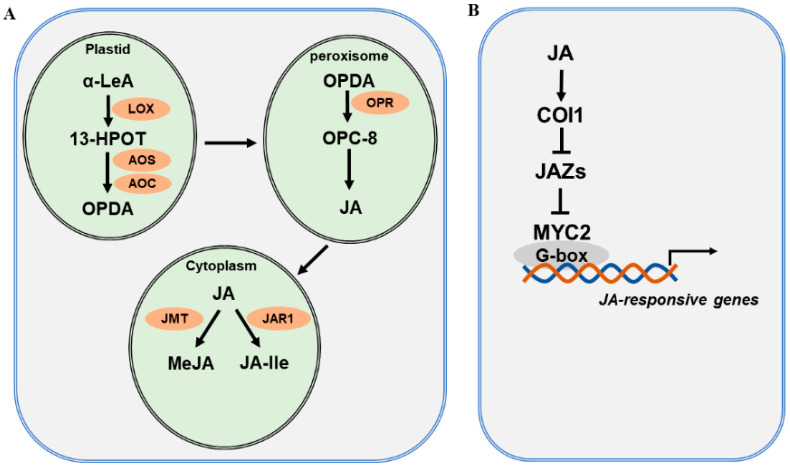
JA biosynthesis and signaling pathway. (**A**) A simplified JA biosynthesis pathway from α-linolenic acid (α-LeA). JA and its derivatives are produced from α-LeA through several sequential steps, which are catalyzed by lipoxygenase (LOX), allene oxide synthase (AOS), and allene oxide cyclase (AOC) in plastids; OPDA reductase (OPR) in peroxisomes; and jasmonate-amido synthetase (JAR1) and jasmonate methyl transferase (JMT) in the cytoplasm. (**B**) A simplified JA signaling pathway. Three main components are involved in JA signaling: the COI receptor, the JAZ repressor, and the MYC2 transcription factor.

The JA biosynthetic pathway and the major enzymes involved have been well characterized and extensively reviewed [27,29,44,45,46,47,48]. JA biosynthesis starts with polyunsaturated fatty acids released from plastid membranes through the action of phospholipase (PLA) [49]. Current evidence supports the assertion that JA is derived via the α-linolenic acid (α-LeA, C18:3) pathway and the hexadecatrienoic acid (HTA, C16:3) pathway [50]. As the α-LeA pathway is dominant in the biosynthesis of JA, we focus on this pathway to explain JA biosynthesis (Figure 1A). Overall, four major enzymes are engaged in JA biosynthesis from α-LeA, comprising lipoxygenase (LOX), allene oxide synthase (AOS), allene oxide cyclase (AOC), and oxophytodienoic acid reductase (OPR) [51,52]. In plastids, LOX catalyzes the first step of JA biosynthesis. α-LeA is converted to 13(*S*)-hydroperoxy-octadecatrienoic acid (13-HPOT) by LOXs. In Arabidopsis, four LOXs—LOX2, LOX3, LOX4, and LOX6—are able to oxygenate α-LeA [53]. Each LOX may function differentially depending on the types of external stimuli. For instance, LOX6 is predominantly involved in JA production upon wounding and drought stress [54,55]. Subsequently, 13-HPOT is catalyzed by AOS to produce 12,13(*S*)-epoxy-octadecatrienoic acid (12,13-EOT). AOS is a cytochrome P450 enzyme, which uses oxygenated fatty acid hydroperoxide substrates as oxygen donors. There is only one *AOS* gene in Arabidopsis, and mutation in *AOS* leads to disrupted JA biosynthesis in response to wounding [56]. 12,13-EOT is further converted to 12-oxo-phytodienoic acid (12-OPDA), catalyzed by AOC. Four *AOC* genes have been identified and found to act redundantly in the biosynthesis of JA in Arabidopsis [57]. Next, 12-OPDA is translocated by the transporter COMATOSE (CTS1) to the peroxisome [58]. In the peroxisome, OPDA is reduced by OPDA reductase (OPR) to produce OPC-8:0. OPRs are encoded by six *OPR* genes in the Arabidopsis genome; however, only OPR3 acts on OPDA. OPC-8:0 is then subjected to three rounds of β-oxidation by acyl-CoA oxidase (ACX), L-3-KETOACYLCOA THIOLASE (KAT), and multifunctional protein (MFP) [59]. Finally, JasmonoylCoA, which is generated through β-oxidation reaction, can be further cleaved by THIOESTERASE (TE) to produce (+)-*7-iso*-JA, which is then transported to the cytoplasm. In the cytoplasm, various JA derivatives are formed, including methyl jasmonate (MeJA) and JA-isoleucine (JA-Ile) [60,61]. The conjugation of (+)-*7-iso*-JA with isoleucine produces JA-Ile, and the reaction is catalyzed by jasmonate-amido synthetase (JAR1). The *jar1* mutant was identified as the first JA-insensitive mutant, and the JA-Ile level in mutant plants is severely reduced [62]. The methylation of JA forms MeJA, with catalysis by jasmonate methyl transferase (JMT). JA-Ile and MeJA are active forms of JA, and they can be converted to inactive 12-OH-JA by jasmonate-induced oxygenases (JO) and jasmonic acid oxidases (JAO) [63,64].

The JA signaling pathway has been well defined primarily in Arabidopsis and tomato. In brief, it consists of the receptor CORONATINE INSENSITIVE 1 (COI1), the repressors JASMONATE ZIM-DOMAIN PROTEIN (JAZs), and the master transcription factors MYCs (Figure 1B). Early genetic screens identified *coronatine insensitive 1* (*coi1*), which is insensitive to the functional homolog of JA-Ile, coronatine. Later studies confirmed that COI1 acts as a receptor that perceives active JA [65,66]. COI1 is an F-box protein [67] that is able to associate with SKP1 and CULLIN1 to form an E3 ubiquitin ligase complex, SCF^COI1^. In search of the substrate for SCF^COI1^, researchers from three independent laboratories discovered JASMONATE ZIM-DOMAIN (JAZ) proteins, which are repressors of JA signaling [68,69,70]. JAZs belong to the plant-specific TIFY family, possessing a core TIF[F/Y]XG motif within the ZIM (ZN-FINGER PROTEIN EXPRESSED IN INFLORESCENCE MERISTEM) protein domain. There are 12 JAZ proteins that have been identified in Arabidopsis [68,69,71]. These JAZs are distinct from other proteins in the TIFY family as they contain a C-terminal Jas motif, SLX2FX2KRX2RX5PY [70,72,73]. The interaction of COI1 with the Jas domain of JAZ proteins forms the co-receptor complex [74,75]. TOPLESS (TPL) and TPL-related proteins (TPRs) are corepressors that interact with JAZ proteins through the ETHYLENE RESPONSE FACTOR (ERF)-ASSOCIATED AMPHIPHILIC REPRESSION (EAR) motif. MYC2, a bHLH transcription factor, is the master regulator of JA signaling and mediates a variety of biological processes. MYC2 is repressed by JAZ proteins and is released following the degradation of JAZs [69,75,76]. Eventually, MYC2 activates various downstream JA-responsive genes [44,77,78,79]. MYC2 plays multifaceted roles in growth and development, defense against biotic stress, abiotic stress response, and regulation of secondary metabolite biosynthesis. MYC3 and MYC4 are two close homologs of MYC2. MYC2 forms dimers with MYC3 and MYC4 to modulate the transcription of various target genes by binding to the G-box or its variants within the promoters [80,81].

## 3. The Role of JA in Cold Stress Response

Cold stress generally refers to two types of stresses: chilling stress, with a temperature ranging from 0 °C to 15 °C, and freezing stress, with a temperature below 0 °C. Cold stress is one of the most severe environmental stresses in plants. Cold stress inhibits plant growth and development and threatens crop productivity. To cope with cold stress, plants have evolved a wide variety of mechanisms. JA, a classical phytohormone, positively mediates plant cold response. Plenty of studies have shown that JA production is increased in plants in response to cold stress, which implies the potential role of JA in the response to cold stress. For instance, upon cold stress, JA accumulation is markedly enhanced in Arabidopsis, tomato, and rice [82,83,84,85]. Consistent with increased JA accumulation, the expression of JA biosynthesis genes is induced by low temperatures. As observed in rice, cold stress triggers the expression of *OsLOX2*, *OsAOC*, *OsAOS1*, and *OsAOS2* and promotes endogenous JA levels [85]. Similarly, in *Artemisia annua*, higher levels of JA and increased expression of JA biosynthesis genes were observed following cold treatment [35]. Furthermore, the application of exogenous MeJA potentiates cold tolerance in a variety of species, including banana, tomato, loquat, orange, guava, mango, and peach [24,86,87,88,89,90]. All these results support the assertion that JA is involved in plant cold stress response.

The role of JA in cold response is further substantiated by mutant or transgenic plants with altered JA biosynthesis. Arabidopsis plants with mutations in *AOS* and *LOX2* show impaired JA biosynthesis, and these plants are hypersensitive to low temperatures [82]. Another study showed that MaLBD5 (lateral-organ boundaries domain) is associated with the JA-mediated cold response in banana fruits. MaLBD5 promotes JA biosynthesis by transactivating the expression of *MaAOC2* [91]. Furthermore, a genetic study showed that *HAN1*, a rice gene that encodes an oxidase that catalyzes the active form JA-Ile to the inactive form 12OH-JAIle, negatively regulates cold tolerance [92]. In addition, transgenic Arabidopsis plants overexpressing GLR1.2 (glutamate-like receptor) and GLR1.3 display enhanced accumulation of JA by activating the expression of JA biosynthesis genes and increased cold tolerance [93].

In an attempt to understand the underlying mechanisms of JA-mediated cold tolerance, numerous studies have revealed that major components of the JA signaling pathway play a critical role in cold tolerance in plants. Being the master regulator of JA signaling, MYC2 is of great importance in cold response. In *Poncirus trifoliata*, MYC2 targets a betaine aldehyde dehydrogenase gene (*PtrBADH-l*) and directly upregulates it, thereby increasing the production of glycine betaine. A high level of glycine betaine confers cold tolerance in *Poncirus trifoliata* [94]. In tomato, MYC2 targets and upregulates *ADC1*, which is a putrescine biosynthesis gene, leading to enhanced putrescine accumulation and decreased cold damage [83]. Under cold conditions, MYC2 directly stimulates the expression of *SlGSTU24*, a JA-responsive glutathione S-transferase gene, and consequently alleviates cold-induced oxidative stress [84]. These results indicate that JA positively regulates cold response by promoting the production of antioxidant enzymes and non-enzymatic cryoprotective compounds through MYC2.

The module ICE (inducer of CBF expression)-DREB1/CBF (dehydration-response element-binding protein 1/C-repeat binding factors) plays a vital role in cold response in plants [95,96]. DREB1/CBFs are AP2/ERF (APETALA2/ETHYLENE-RESPONSIVE FACTOR)-type transcription factors capable of binding to DREs (dehydration-responsive element) in the promoters of target genes and acting as key regulators of COR (cold-regulated) genes [97,98,99]. Previous studies have identified three DREB1/CBF genes: *DREB1A/CBF3*, *DREB1B/CBF1*, and *DREB1C/CBF2* [98,100]. Cold stress leads to rapid induction of these genes, and mutations in them severely impair cold tolerance [101,102]. Overexpression of *CBF* genes induces the expression of numerous cold-inducible genes and confers cold tolerance [103,104]. ICE1 is an MYC-like basic helix–loop–helix transcription factor that acts as a master regulator in the DREB1/CBF pathway. In the past two decades, a large number of studies have established the role of ICE1 in the expression of *DREB1/CBF*. However, recently, it has been reported that repression of *CBF3* in *ice1-1* mutant plants is due to DNA-methylation-mediated gene silencing caused by inserted T-DNA, not by *ICE1* mutation, and that ICE1 is not associated with *CBF3* expression [105,106,107].

JAZs, the repressors of JA signaling, are important for the JA-mediated cold response, and the ICE-CBF module is involved in this process. In Arabidopsis, JAZs interact with ICEs to repress the expression of *CBFs*. Upon cold treatment, JA accumulation is increased, promoting the degradation of JAZs, thus releasing ICEs. ICEs then activate *CBFs*, conferring cold tolerance in Arabidopsis [82]. In apple, MdJAZ1 and MdJAZ2 interact with the transcription factor BBX37 and suppress the transcription of *MdCBF1* and *MdCBF4*. In response to cold stress, increased JA leads to the degradation of MdJAZ1 and MdJAZ2, allowing BBX37 to activate *MdCBF1* and *MdCBF4* [108]. Interestingly, under cold stress, the expression of *MaMYC2a and MaMYC2b* is tremendously induced in banana, and MaMYC2 physically interacts with MaICE1, thus triggering the expression of *MaCBF1* and *MaCBF2* [87]. In Arabidopsis, SFR6 (SENSITIVE TO FREEZING 6) controls the expression of cold-regulated genes by acting on the CBF module [109,110,111]. Meanwhile, SFR6 is also involved in the regulation of JA responses [112,113]. These studies highlight the role of JA in cold response via the induction of CBFs.

## 4. The Role of JA in Heat Stress Response

Temperatures that exceed the physiological optimum ranges for growth and development may cause heat stress in plants. Heat stress is one of the most devastating abiotic stresses, leading to impaired cell homeostasis [114,115] and stunted growth and development in plants. As global warming continues, high temperatures are occurring more frequently, posing a threat to crop yields and agricultural sustainability [116]. To survive under heat conditions, plants have developed various mechanisms, among which phytohormones play a vital role.

Recently, JA has emerged as a key player in the alleviation of adverse effects caused by environmental stresses. Several studies have pointed out the role of JA in the response to heat stress in plants. Heat stress increases the accumulation of a range of jasmonates, including jasmonic acid, 12-oxophytodienoic acid (OPDA), and a JA-isoleucine (JA-Ile) conjugate in Arabidopsis. Exogenous application of MeJA maintains cell viability and confers tolerance to heat stress [117]. A genetic study demonstrated that an Arabidopsis mutant with constitutive activation of JA signaling exhibited enhanced heat tolerance, whereas Arabidopsis plants lacking the JA signaling pathway displayed impaired heat tolerance [117]. In another study, heat shock boosted the JA signaling pathway and promoted JA production in agarwood [118]. Under heat stress, the expression of *AtOPR3* is activated, leading to increased JA biosynthesis and accumulation in Arabidopsis. Then, the JA signaling pathway activates *DREB2A* expression, thus enhancing heat tolerance [119]. These results demonstrate that JA plays an important role in heat stress response. However, there are studies that do not support a positive role of JA in heat tolerance. In an effort to uncover how different abiotic stresses affect JA levels in rice, researchers found that the JA level was reduced by heat stress, while it was increased by drought and cold stress. Consistently, genes involved in JA biosynthesis were suppressed by heat stress but were induced by drought and cold stress [85]. Furthermore, a recent study showed that high temperature leads to a notable reduction in the levels of the active form of JA as a result of increased expression of *JOXs* and *ST2A* in Arabidopsis [120]. In cotton, a high temperature decreases the expression of *GhAOC2* in anthers, leading to reduced JA biosynthesis [42]. Notably, the expression of *HSPs* (heat shock proteins), which are markers for heat tolerance, is recalcitrant to exogenous JA or altered JA signaling mutants, complicating the associations of JA with heat tolerance [115]. These seemingly contradictory observations on the role of JA in heat response may be ascribed to differences in the species, experimental settings, or growth temperatures used in various studies.

HEAT SHOCK PROTEINS (HSPs) function positively in heat tolerance by preventing protein denaturation and refolding damaged proteins [121]. SUPPRESSOR OF G2 ALLELE OF SKP1 (SGT1) protein acts as a cofactor of HSP90 in both plants and mammals. SGT1 and HSP90 can form functional complexes that impart greater heat tolerance. Genetic and biochemical studies have revealed that SGT1 is involved in JA signaling by maintaining the steady state of COI1 [122]. Suppression of HSP90 in RNAi plants decreased the expression of JA marker genes, and the application of HSP inhibitor reduced the expression of COR-regulated genes, suggesting a crucial role of HSP in JA signaling and heat response.

WRKY transcription factors constitute a large WRKY superfamily. In Arabidopsis, 74 WRKY members have been identified [123,124]. WRKY transcription factors function in the regulation of multiple biological processes, including plant growth, development, and stress response [125]. Some WRKY members are involved in heat response, such as AtWRKY25, AtWRKY39, and OsWRKY11 [125,126,127,128] (Li et al., 2010). In pepper plants, CaWRKY40 was induced by heat stress and exogenous JA. Overexpression of CaWRKY40 derepressed the JA biosynthesis gene *LOX1* under heat stress [129]. These results indicate that JA regulates the expression of *CaWRKY40*, which then promotes the expression of heat-responsive genes and confers heat tolerance in pepper plants.

## 5. Crosstalk between JA and Other Phytohormones during the Response to Temperature Stress

### 5.1. Crosstalk between JA and Other Phytohormones in the Regulation of Cold Stress Response

**Figure 2:**
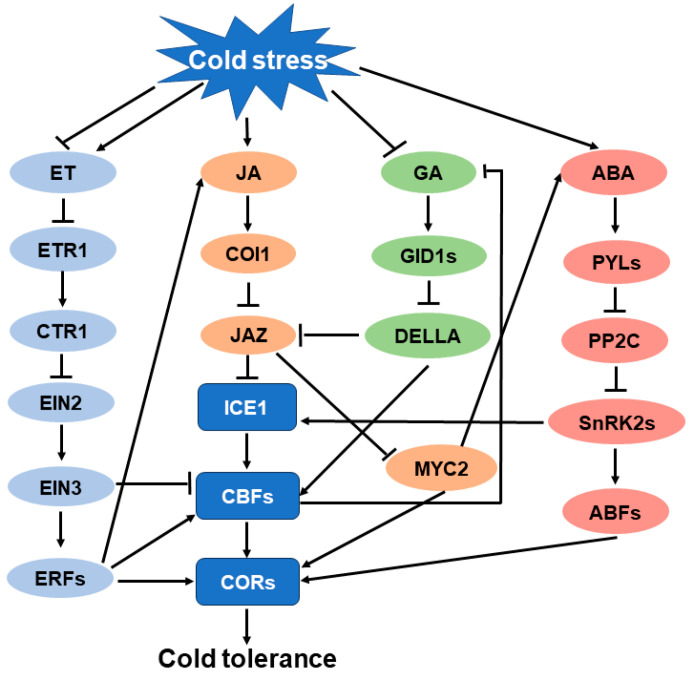
Schematic representation of JA crosstalk with ET, GA, and ABA in cold stress response. Arrows indicate activation process and bars indicate inhibition process.

Recent studies have shown that in most cases, JA does not act alone but interacts with other phytohormones during cold stress responses. It has been demonstrated that JA functions synergistically with ABA and ET in the regulation of cold tolerance; however, JA acts antagonistically with GA during cold stress response. Summarized in Table 1 are case studies showing the crosstalk between JA and other phytohormones in the regulation of cold stress response. Key components that are involved in in the crosstalk between JA and other phytohormones are shown in Figure 2.

#### 5.1.1. Crosstalk between JA and ABA in the Regulation of Cold Stress Response

Abscisic acid (ABA) is an essential hormone that plays multiple roles in growth, development, and stress responses. It is not only a primary hormone for drought response but also a key hormone regulating cold response in plants. Like JA, cold stress increases endogenous ABA accumulation in plants, and exogenous application of ABA promotes cold tolerance in a variety of species [130,131,132,133,142,143]. Both JA and ABA are involved in the regulation of cold stress response, suggestive of the potential crosstalk between them during cold stress. Recently, several studies have demonstrated an association of JA with ABA in response to cold stress. A study showed that JA acts downstream of ABA to activate the expression of *CBFs* to confer cold tolerance in tomato [34]. The authors drew this conclusion based on the observation that exogenous MeJA improved the cold tolerance of an ABA-deficient mutant, while exogenous ABA failed to improve the cold tolerance of a JA-deficient mutant. In line with this observation, ABA was capable of inducing the expression of JA biosynthesis genes and eventually increased JA accumulation. Intriguingly, contrary to these results, a recent work reported that JA-induced cold tolerance is partially dependent on ABA in tomato seedlings [133]. The authors provided several lines of evidence to support their conclusion. Firstly, JA increased endogenous ABA accumulation under cold stress; secondly, blocking ABA biosynthesis attenuated JA-induced cold tolerance; lastly, MYC2 in the JA signaling pathway was able to target the promoter of *NCED2*, an ABA biosynthesis gene, and activate its expression. These results suggest complicated crosstalk between JA and ABA in cold response; further studies are necessary to clarify their relationship.

#### 5.1.2. Crosstalk between JA and Ethylene in the Regulation of Cold Stress Response

Ethylene is another phytohormone that mediates stress response in plants. Initially identified as a plant hormone associated with ripening, ethylene also plays a vital role in biotic and abiotic stress response. Several studies have shown that ethylene is involved in the response to cold stress, since ethylene production is tremendously increased in response to cold stress in various species, including tomato (*Solanum lycopersicum*) [134], grapevine (*Vitis vinifera*) [135], winter rye (*Secale cereale*) [136], apple (*Malus × domestica*) [137], and avocado fruit (*Persea americana*) [138]. Though both JA and ethylene regulate cold stress response, how they interact during cold stress has not been well studied. OsERF096, a downstream transcription factor in the ethylene signaling pathway, represses the JA-mediated cold signaling pathway, thus negatively regulating cold tolerance in rice [137]. JAZ proteins, which function as repressors of the JA signaling pathway, are able to physically interact with EIN3, a key regulator in the ethylene signaling pathway, and suppress the transcriptional activity of EIN3 [82,144]. More recently, a study showed that the ethylene response factor gene *SlERF.B8* is induced by both JA and cold stress, and SlERF.B8 activates the JA biosynthesis genes and enhances JA accumulation under cold conditions. These studies indicate that JA interacts with ethylene in cold stress response.

#### 5.1.3. Crosstalk between JA and Gibberellin in the Regulation of Cold Stress Response

Unlike JA, ABA, and ethylene, gibberellin (GA) negatively regulates plant response to low-temperature stress. Mutations in DELLA genes, repressors of the GA signaling pathway, lead to enhanced sensitivity to cold stress [145]; however, a GA-deficient mutant exhibits increased cold tolerance [139,146]. Interestingly, DELLA proteins physically interact with JAZ proteins, allowing the integration of GA and JA signaling [140]. DELLA proteins competitively bind to MYC2 with JAZ proteins. Without GA, DELLA proteins compete with MYC2 to bind to JAZs, stimulating MYC2 to activate JA-responsive gene expression. In the presence of GA, DELLAs are degraded, promoting the binding of JAZ to MYC2; as a consequence, JA signaling is attenuated. It is thus possible that GA negatively regulates cold response by suppressing JA signaling. Future efforts are needed to unravel the molecular basis of JA and GA crosstalk in plant cold response.

#### 5.1.4. Crosstalk between JA and Melatonin in the Regulation of Cold Stress Response

It is interesting to note that in addition to classical phytohormones, JA also appears to interact with some new plant growth regulators, such as melatonin. Melatonin, which is a typical hormone in animals and humans, was recently identified as an important growth regulator in plants. The identification of the receptor PMTR1 rendered melatonin a potential plant hormone [147,148]. In the past decade, numerous studies have supported the role of melatonin in the regulation of plant growth and stress responses, including cold response. The role of melatonin in modulating cold tolerance has been established in several species, such as tomato, Arabidopsis, and tea plants [11,149,150]. Interestingly, a recent study supports the assertion that melatonin interacts with JA during cold stress response [24]. In tomato, melatonin and JA have synergistic effects on cold tolerance. The production of melatonin and JA was found to be remarkably induced by cold stress, and their exogenous application stimulated cold tolerance. Importantly, blocking melatonin biosynthesis decreased the JA-mediated cold stress response, and inhibiting JA biosynthesis resulted in reduced melatonin biosynthesis. Furthermore, when JA signaling was attenuated via the downregulation of MYC2, melatonin accumulation was decreased under cold stress. Similar results were also obtained in a study on grated watermelon cold tolerance [141]. These results confirm the interaction between melatonin and JA in the modulation of cold tolerance in plants. However, the exact mechanisms underlying the crosstalk between melatonin and JA are largely unknown and warrant further investigations.

### 5.2. Crosstalk between JA and Ethylene in Regulating Heat Stress Response

In the past decades, it has been well established that JAs and ethylene often act cooperatively in the regulation of defense against biotic stresses in plants. Recent studies have shown that they also play vital roles in heat stress response. A large body of studies demonstrated that heat stress regulates the levels and signaling of both JA and ethylene, making it possible that the two phytohormones may interact to modulate heat tolerance in plants. Unexpectedly, in some cases, JA and ethylene antagonistically regulate heat stress response [151,152]. Arabidopsis mutants with impaired JA biosynthesis or JA signaling display hypersensitivity to heat stress, whereas mutation in *EIN2*, the central regulator of ethylene signaling, confers greater heat tolerance, as EIN2 plays a negative role in heat tolerance [115,117]. However, there are studies demonstrating that *ein2* and *ein3eil1* mutants with impaired ethylene signaling show attenuated heat tolerance [153,154]. The interaction of JA and ethylene has also been observed during ethylene biosynthesis. A study showed that JA induces ethylene biosynthesis by improving the activities of ethylene biosynthesis enzymes in tomato [155]. The major interacting point between JA and ethylene is EIN3, the key transcription factor in the ethylene signaling pathway [156,157,158].

## 6. Conclusions and Future Perspectives

With global climate change, extreme temperatures are occurring more frequently than ever before, posing a serious threat to plant growth and crop yields. In recent decades, there has been increasing evidence that JA plays a vital role in temperature stress response in plants. Key constituents in the JA signaling pathway have been uncovered, and some of them have been confirmed as important regulators in the response to temperature stress; however, at the molecular level, our understanding of JA and JA signaling in association with temperature stress remains rudimentary and warrants further studies. Furthermore, though the role of JA in cold stress response is well established, the role of JA in heat stress response is still under debate. Depending on the species, experimental setting, and temperature range, the role of JA in heat tolerance can be either positive or negative. It is therefore important to determine external and internal factors that would explain this discrepancy, and it is necessary to perform more in-depth analyses to validate the positive or negative role of JA in the response to heat stress.

As a versatile plant hormone, JA has been documented to crosstalk with several other hormones. The JA signaling pathway is well conserved across species; however, depending on the species, novel regulators may exist to integrate JA signaling with other signaling pathways in order to better adapt to specific temperature conditions. Thus, future studies should be directed to identify novel regulators that connect the JA signaling pathway and cold response, and to explore new mechanisms underlying the crosstalk between JA and other hormones in response to temperature response. Such studies will expand our understanding of how JA signaling is integrated into other stress signaling networks in temperature stress response.

## Figures and Tables

**Table 1:** Case studies showing the crosstalk between JA and other phytohormones during cold stress response.

Phytohormones	Species	References
ABA	*Arabidopsis thaliana*	Lång et al. [130]
ABA	*Arabidopsis thaliana*	Mäntyla et al. [131]
ABA	*Cucumis melo*	Li et al. [132]
ABA	*Solanum lycopersicum*	Ding et al. [133]
ABA	*Solanum lycopersicum*	Wang et al. [34]
Ethylene	*Solanum lycopersicum*	Dong et al. [134]
Ethylene	*Vitis vinifera*	Sun et al. [135]
Ethylene	*Secale cereale*	Yu et al. [136]
Ethylene	*Malus × domestica*	Wang et al. [137]
Ethylene	*Persea americana*	Hershkovitz et al. [138]
GA	*Arabidopsis thaliana*	Richter et al. [139]
GA	*Arabidopsis thaliana*	Hou et al. [140]
Melatonin	*Solanum lycopersicum*	Ding et al. [24]
Melatonin	*Citrullus lanatus*	Li et al. [141]

## Data Availability

No new data were created or analyzed in this study. Data sharing is not applicable to this review.
